# Impact of intravenous immunoglobulin treatment on peripheral blood cells in children with Kawasaki disease complicated with coronary artery lesion

**DOI:** 10.1186/s13052-025-01891-2

**Published:** 2025-02-11

**Authors:** Junshan Zhao, Yingying Ma, Li Xin, Lijun Wang, Hongliang Gao

**Affiliations:** Department of Intensive Care Medicine Division 2, Hebei Children’s Hospital, Shijiazhuang City, 050031 China

**Keywords:** Intravenous immunoglobulin, Coronary arteries, Kawasaki disease, Peripheral blood cells

## Abstract

**Background:**

Kawasaki disease (KD) primarily affects young children and can lead to coronary artery lesions. Intravenous immunoglobulin (IVIG) treatment is essential; however, it may fail in 10-20% of cases, increasing the risk of complications. Complete blood count (CBC) tests can help assess disease severity and predict risks. This study investigated the impact of IVIG on peripheral blood cells, including neutrophil count, platelet-lymphocyte ratio, hemoglobin level, mean platelet volume (MPV), erythrocyte sedimentation rate (ESR), and C-reactive protein (CRP), in children with KD complicated by coronary artery lesions (CALs).

**Methods:**

This retrospective analysis included children diagnosed with typical KD. The neutrophil count, hemoglobin level, platelet-lymphocyte ratio, MPV, ESR, and CRP were compared between those with (CAL^+^) and without (CAL^−^) CALs during the acute phase, as well as at 7 days, 1 month, and 2 months after normalizing body temperature with IVIG.

**Results:**

A total of 76 children with KD were analyzed, including 30 with CAL^+^. CAL^+^ and CAL^−^ patients exhibited elevated neutrophil counts during the acute phase, with no significant differences (*P* > 0.05) between groups. After IVIG treatment, CAL^−^ patients demonstrated a more pronounced reduction in neutrophil count (*P* < 0.05) than CAL^+^ patients. Hemoglobin levels differed significantly during the acute phase (*P* < 0.05) but were comparable post-treatment (*P* > 0.05) between CAL^+^ and CAL^−^ patients. The platelet-lymphocyte ratio varied significantly between groups during the acute phase and 1-month post-treatment (*P* < 0.05). Mean ESR and CRP levels were significantly elevated at all time points in the CAL^+^ group compared with the CAL^−^ group. No significant differences in MPV were observed between groups.

**Conclusions:**

After IVIG treatment, CAL^−^ patients demonstrated a more important reduction in neutrophil count than CAL^+^ patients after IVIG. Pediatric patients with KD and CAL^+^ showed lower hemoglobin and platelet-lymphocyte ratio and higher ESR and CRP compared with CAL^−^, suggesting that they may serve as indicators for CAL in pediatric patients with KD.

## Background

Kawasaki disease (KD) is an acute systemic vasculitis affecting small- and medium-sized arteries with unknown etiology and was first reported by a Japanese physician, Tomisaku Kawasaki, in 1967 [1, 2]. The incidence of KD has been increasing annually, particularly in Japan, Korea, Taiwan, and China [3]. It primarily affects children under 5 years old, with a higher incidence in males than females [4]. Notably, approximately 25% of KD patients develop coronary artery lesions (CAL), including coronary artery aneurysms (CAA) [5]. High-dose intravenous immunoglobulin (IVIG) combined with aspirin therapy can reduce the incidence of CAL in KD patients to below 5%. However, 10-20% of KD patients show poor response to IVIG combined with aspirin treatment [6], and those who do not respond to IVIG are at significantly higher risk of CAL development compared to IVIG responders [7]. KD-induced coronary artery damage is a major cause of acquired heart disease in children [8]. Yet, the pathogenesis of KD remains unclear, with most researchers attributing its occurrence to immune dysregulation [9], although the precise mechanisms underlying immune dysregulation remain poorly understood.

Complete blood count (CBC) is a routine blood test commonly used to find overall health and a wide range of clinical conditions due to its simplicity, high efficiency, low cost, and high patient compliance. Peripheral blood cell analysis can be used to determine KD severity. Systemic inflammation occurs during the onset and progression of KD, and fluctuating inflammatory markers of blood may be used to assess the inflammation precisely in KD. Laboratory parameters may be used in identifying patients with KD who are at greater risk of CAA development after IVIG treatment. The fluctuations in the risk factors compared with baseline values might possess greater predictive power for CAA development in patients with KD [10]. The high neutrophil-albumen ratio was correlated to older age, high WBC, neutrophil percentage, ALT, total bilirubin, and C-reactive protein (CRP), as well as with high incidence of IVIG-resistance, and low hemoglobin, platelet count, ALB, and sodium level in KD [11]. The erythrocyte sedimentation rate (ESR) and CRP are important inflammatory indicators and play a significant role in diagnosing and managing Kawasaki disease [12–14]. A previous study showed that CRP levels were independently associated with CAL^+^ in children with KD, while ESR was not [15].

This retrospective study aimed to investigate the impact of IVIG treatment on peripheral blood cells, including neutrophil granulocyte (NE) count, hemoglobin (Hb) levels, platelet-to-lymphocyte ratio (PLR), mean platelet volume (MPV), ESR, and CRP, in children with KD, complicated by coronary artery lesions.

## Methods

### Study design and participants

A retrospective analysis was conducted on 76 confirmed consecutive cases of typical KD at Hebei Children’s Hospital from January 2021 to December 2023. The diagnostic criteria followed the American Heart Association (AHA) Kawasaki Disease Diagnostic Guidelines (2017 edition) [16]. The inclusion criteria were as follows: (1) children with a diagnosis of complete KD; CKD was diagnosed by a fever of ≥ 5 days and at least 4 of 5 clinical features, namely non-exudative conjunctivitis, oropharyngeal lesions, acute non-suppurative cervical lymph node enlargement, pleomorphic rash, including redness, and abnormalities at the extremities with or without coronary artery lesions; (2) children who had good physical health before the onset of KD; (3) children who had no other severe underlying diseases, such as immunodeficiency disease, familial genetic disease, congenital metabolic disease, congenital heart disease, or other basic diseases before the onset of KD; (4) children with no recent history of infectious diseases, surgeries, and blood transfusions. The exclusion criteria were as follows: (1) children who used medications during the diagnosis and treatment of KD that could have affected the target laboratory tests (e.g., glucocorticoids, aminopyrine, betamethasone, tetracycline antibiotics, and β-lactam antibiotics); (2) children who did not have consent or had withdrawal of consent by legal guardians; (3) children with incomplete or missing data. Legal guardians of participating children provided informed consent, and the protocol for the study was approved by the hospital’s Medical Ethics Committee. The included patients were divided according to the presence (CAL^+^ group) or absence (CAL^−^ group) of coronary artery lesions based on the echocardiographic findings. The diagnostic criteria of CAL by echocardiography were the presence of any of the following three features: (1) z score *≥* 2.5 for the left anterior descending coronary artery (LAD) or right coronary artery (RCA), (2) coronary artery aneurysm, and 3) *≥* 3 other suggestive features including decreased left ventricular function, mitral regurgitation, pericardial effusion, or z scores of 2-2.5 for the LAD or RCA [17].

All KD children included in the study were given IVIG combined with aspirin and other standard treatment in the acute stage (fever time 5–10 days). IVIG dosages were 2 g/kg, one-time intravenous infusion within 12 h for children weighing < 20.0 kg and 1 g/kg•d, and continuous use for 2 days for children weighing ≥ 20.0 kg. The aspirin dose was 30–50 mg/kg•d, divided into three oral administrations and 3–5 mg/kg 72 h after the normal body temperature.

### Data collection and definitions

Participants were classified into the CAL^+^ and CAL^−^ groups based on echocardiographic evidence of coronary artery lesions. Data collection included routine blood parameters, C-reactive protein (CRP), and erythrocyte sedimentation rate (ESR) during the acute phase of KD, 7 days after normal body temperature restoration post-IVIG treatment, and at 1 month and 2 months post-IVIG treatment.

### Statistical analysis

The data were analyzed using the statistical software SPSS 20.0. Metric data conforming to a normal distribution are presented as mean ± standard deviation. Student t-test and one-way analysis of variance (ANOVA) were used to compare the variables between the two groups, where *P* < 0.05 indicated statistical significance.

## Results

### Comparison of neutrophil count

The CAL^+^ group comprised 30 cases (19 males and 11 females) aged between 1 year and 8 years. The CAL^-^ group consisted of 46 cases (27 males and 19 females) aged between 1 and 9 years. There were no statistically significant differences in age or sex between the two groups (*P* > 0.05). In children with KD, the neutrophil count was significantly elevated in acute phases of CAL^+^ and CAL^-^ groups, with no statistical significance observed between the groups (14.15 ± 8.21 vs. 11.79 ± 1.52, *P* > 0.05). Following normalization of body temperature, at 7 days and 1 month post-IVIG treatment, neutrophil count in both groups significantly decreased to normal, with the CAL^-^ group showing a more pronounced statistically significant reduction than the CAL^+^ group (*P* < 0.05). At 2 months post-IVIG treatment, neutrophil count was still decreased to normal levels but without significant differences between the two groups (*P* > 0.05) (Table 1).

**Table 1:** Comparison of neutrophil count between the CAL^+^ and CAL^−^ groups, (mean ± SD), (10^9^/L)

Group	*N*	Acute phase	7 days post IVIG-induced normothermia	1 month post-IVIG treatment	2 months post-IVIG treatment
CAL^+^ group	30	14.15 ± 8.21	3.56 ± 2.49	3.35 ± 2.49	3.28 ± 1.28
CAL- group	46	11.79 ± 1.52	2.32 ± 1.54	2.72 ± 1.35	3.05 ± 1.93
F Value		1.187	14.211	4.567	0.025
P-Value		0.279	< 0.001	0.036	0.875

IVIG: intravenous immunoglobulin; CAL: coronary artery lesion

### Comparison of platelet-lymphocyte count

**Fig. 1:**
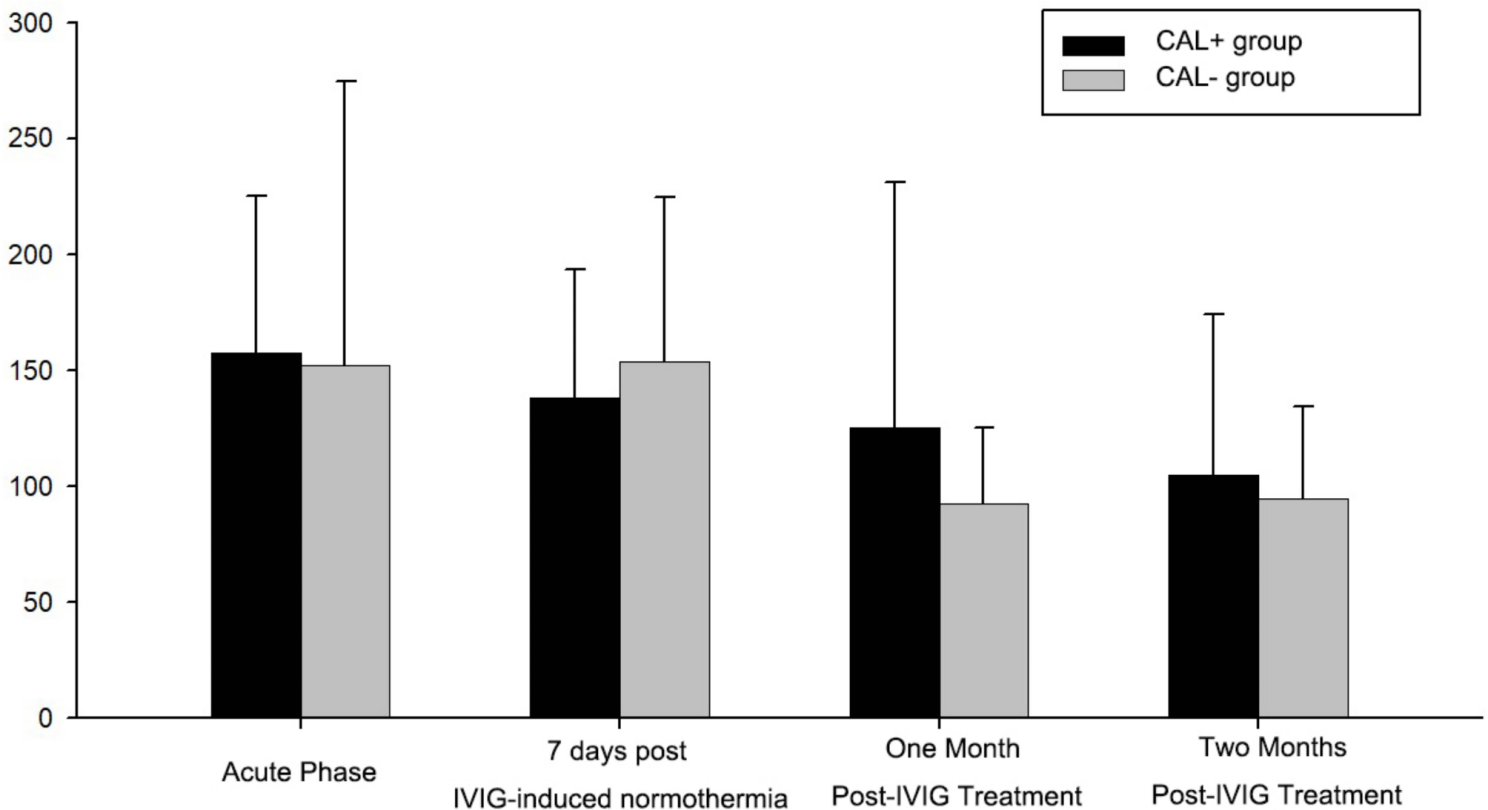
Comparison of platelet-lymphocyte ratio between the CAL^+^ and CAL^−^ groups

The platelet-lymphocyte count comparisons between the CAL^+^ and CAL^-^ groups before IVIG treatment, at 7 days, 1 month, and 2 months after IVIG treatment of normalizing body temperature revealed significant differences. Before IVIG treatment (157.78 ± 67.77 vs. 152.27 ± 122.59; P-value = 0.045), and at 1 month after IVIG treatment (125.17 ± 205.97 vs. 92.64 ± 32.58; P-value = 0.023), platelet-lymphocyte count differed with statistical significance (*P* < 0.05). However, there were no significant differences in platelet-lymphocyte count between the two groups at 7 days after IVIG treatment (138.18 ± 55.29 vs. 153.77 ± 71.26; P-value = 0.440) and at 2 months after IVIG treatment (104.81 ± 69.61 vs. 94.85 ± 39.71; P-value = 0.356) (Fig. 1).

### Comparison of hemoglobin level

**Fig. 2:**
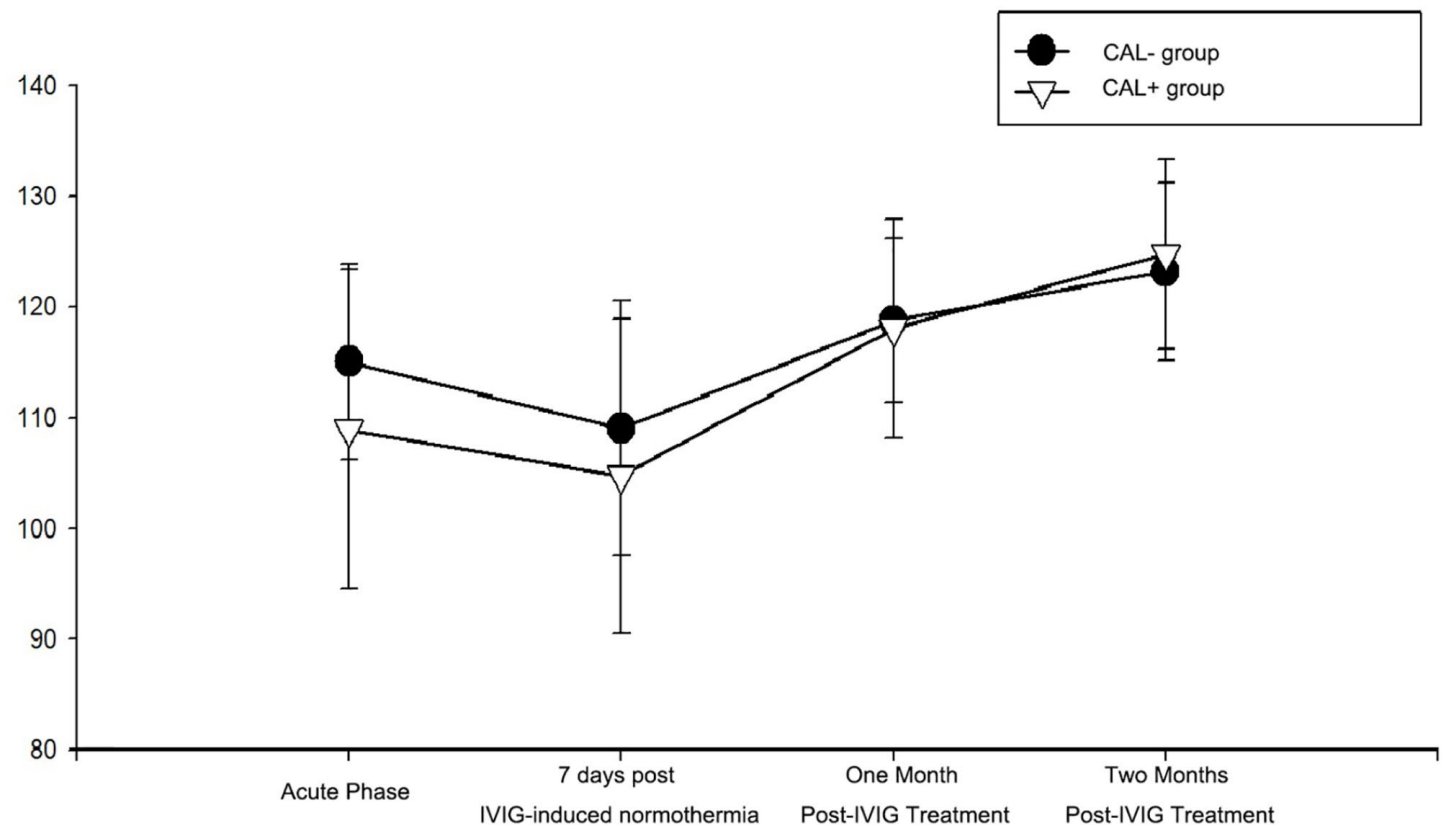
Comparison of hemoglobin levels between the CAL^+^ and CAL^−^ groups

Comparison of hemoglobin levels between the CAL^+^ and CAL^-^ groups showed significant differences during the acute phase before IVIG treatment (108.97 ± 14.37 vs. 115.09 ± 8.83; P-value = 0.030). However, there were no statistically significant differences at 7 days, 1 month, and 2 months after the IVIG treatment (*P* > 0.05) (Fig. 2).

### Comparison of mean platelet volume

**Fig. 3:**
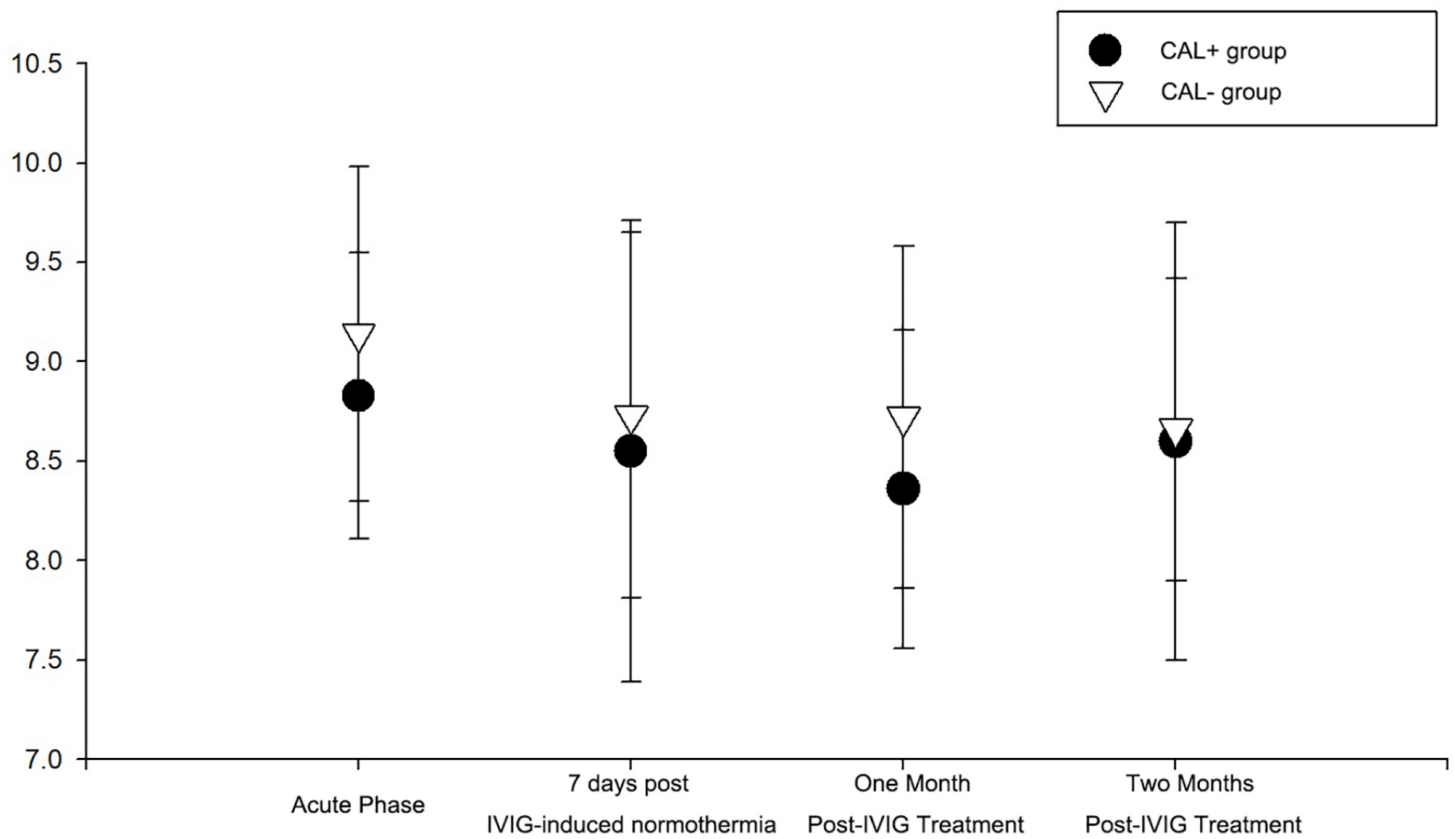
Comparison of mean platelet volume between the CAL^+^ and CAL^−^ groups

Mean platelet volume showed no statistically significant differences during the acute phase before IVIG treatment and at 7 days, 1 month, and 2 months after treatment (*P* > 0.05) between the CAL^+^ and CAL^-^ groups. Additionally, dynamic comparisons within each group across these time points did not statistically differ (*P* > 0.05) (Fig. 3).

### Comparison of C-reactive protein

Mean CRP levels were significantly elevated in the CAL^+^ groups compared with the CAL^-^ group during the acute phase before IVIG treatment and at 7 days, 1 month, and 2 months after treatment (all *P* < 0.001) (Table 2).

**Table 2:** Comparison of CRP between the CAL^+^ and CAL^−^ groups, (mean ± SD), (10^9^/L)

Group	*N*	Acute phase	7 days post IVIG-induced normothermia	1 month post-IVIG treatment	2 months post-IVIG treatment
CAL^+^ Group	30	91.39 ± 22.50	23.82 ± 2.58	5.71 ± 1.41	1.32 ± 0.30
CAL- Group	46	63.96 ± 25.94	15.78 ± 5.43	3.52 ± 1.37	0.90 ± 0.39
F Value		22.478	38.904	45.914	23.626
P-Value		< 0.001	< 0.001	< 0.001	< 0.001

IVIG: intravenous immunoglobulin; CAL: coronary artery lesion

### Comparison of erythrocyte sedimentation rate

Mean ESR levels were significantly elevated in the CAL^+^ groups compared with the CAL^-^ group during the acute phase before IVIG treatment and at 7 days, 1 month, and 2 months after treatment (all *P* < 0.05) (Table 3).

**Table 3:** Comparison of erythrocyte sedimentation rate between the CAL^+^ and CAL^−^ groups, (mean ± SD), (10^9^/L)

Group	*N*	Acute phase	7 days post IVIG-induced normothermia	1 month post-IVIG treatment	2 months post-IVIG treatment
CAL^+^ group	30	82.07 ± 13.33	111.20 ± 10.34	38.13 ± 9.55	15.93 ± 3.96
CAL- group	46	55.50 ± 17.87	80.91 ± 23.79	31.87 ± 10.79	11.91 ± 5.06
F Value		48.590	43.141	6.686	13.527
P-Value		< 0.001	< 0.001	0.012	0.001

IVIG: intravenous immunoglobulin; CAL: coronary artery lesion

## Discussion

KD is an acute systematic vasculitis of children with an immune origin; however, its pathogenesis remains unclear [18]. In this study, neutrophil counts decreased in CAL^+^ and CAL^−^ groups with a more pronounced statistically significant decrease in the CAL^−^ after IVIG treatment. Hemoglobin levels significantly differed between the groups in the acute phase. PLR significantly varied between the two groups during the acute phase and 1 month after IVIG treatment. MPV didn’t statistically differ between the groups at any of the periods investigated.

Immune dysfunction triggered by acute inflammation may play a significant role in KD pathogenesis [16, 19]. Blood routine tests, as common diagnostic tools for inflammatory diseases, are advantageous for determining KD severity due to their simplistic procedures, short turn-around time, data reliability, and good patient compliance [20]. Neutrophil count, an important variable in blood routine tests, precisely reflects the body’s inflammatory response [21]. On autopsies of KD cases after the sudden death of children during the acute phase, neutrophil infiltration in the coronary arteries, rapid formation of large coronary artery aneurysms (CAA), and sudden rupture of CAA were noticed, highlighting the critical role of neutrophils in early coronary artery damage in KD [22]. High levels of anti-elastase-positive neutrophils were observed in the coronary intima during autopsies of KD cases, with peak levels around day 10 of illness onset, providing strong evidence for neutrophil involvement in CAL formation [23, 24]. During the acute phase of KD, intense inflammatory responses prompted neutrophils to migrate to vascular endothelium by the chemotaxis of inflammatory factors, releasing inflammatory mediators that induced endothelial cell apoptosis, necrosis, and disruption of vascular barriers [25]. Neutrophils release enzymes such as cysteinyl aspartate specific proteinase (caspase) and matrix metalloproteinases (MMPs), damaging vascular wall elastic collagen fibers, thereby disrupting vascular integrity and leading to CAL and even CAA [26, 27]. Neutrophils, CRP, and total bilirubin were identified as significant predictors of IVIG treatment resistance in children with KD [28]. In this study, neutrophils significantly increased during the acute phase of KD, with no significant difference between patients with and without CAL. IVIG treatment caused a rapid decrease in neutrophil count, with a more pronounced reduction in the non-CAL group. It suggests that IVIG treatment has a low response to abnormal neutrophil count in the presence of CAL, leading to sustained neutrophil-mediated endothelial cell and vascular wall damage, thus further aggravating the CAL. Therefore, abnormality in neutrophil count can serve as an indicator of the risk of CAL occurrence and a response marker for the efficacy of IVIG treatment.

Platelets play a crucial role in systemic inflammation and immune response. During inflammation, platelets release cytokines, chemokines, and other factors that induce endothelial cells to release inflammatory mediators and promote monocyte adhesion and migration, exacerbating the inflammatory response [29, 30]. Mean platelet volume (MPV) is a measure of platelet activation, reflecting platelet function, infections, diffuse inflammatory reactions, and other conditions [31]. Increased blood platelet counts and platelet activation are the characteristics of KD, which is associated with an increased risk of developing resistance to IVIG treatment and coronary artery lesions. KD patients often exhibit elevated platelet counts, contributing to immune dysfunction and increasing the risk of coronary artery damage. Lymphocytes play a role in immune regulation. The platelet-to-lymphocyte ratio (PLR) reflects the balance between platelet and lymphocyte counts, serving as a biomarker for inflammatory responses and immune system stability. It is closely associated with the degree of coronary artery stenosis and the occurrence of cardiovascular events, making it an important indicator for predicting cardiovascular events [20, 32]. Children with KD have increased leukocyte-platelet aggregates during the acute phase of the disease, and this parameter can be used as a biomarker for disease severity [33]. In this analysis, PLR was higher in KD patients with CAL during the acute phase of KD and 1 month after IVIG treatment. Early elevation of PLR in KD patients indicates a strong inflammatory response and immune system dysregulation, which may be the risk factors for developing CAL. The elevated PLR in patients with CAL after IVIG treatment indicates inadequate IVIG treatment efficacy. This persistent condition causes continuous damage to coronary arteries, endothelial cells, and elastic collagen fibers in the vessel wall. PLR levels correlate positively with the risk of cardiovascular events. During the acute phase of the disease, there is mild fibrous tissue proliferation within the coronary vessel wall, and changes in diameter are not obvious. After four weeks, partial narrowing of the coronary arteries occurs, significantly increasing the risk of cardiovascular damage. These findings align with Li et al.’s conclusion [32]. No significant changes in MPV during disease progression and no significant difference between KD patients with and without CAL are consistent with the findings of Bozlu et al. and Liu et al. [32, 34]. Therefore, PLR might be a sensitive risk factor for CAL and a good indicator for evaluating IVIG treatment efficacy, whereas MPV is not.

In clinical practice, hemoglobin levels decrease during infections and systemic inflammatory responses, possibly due to increased catabolism and suppressed bone marrow hematopoiesis [35]. Children with KD often exhibit decreased Hb levels, and decreased levels are associated with IVIG treatment resistance, although the specific mechanisms remain unclear [36]. Previous studies found that low Hb reduced the oxygen-carrying capacity of the circulatory system in KD patients, leading to tissue hypoxia. The hypoxia triggered sympathetic nervous system activation and excessive renin-angiotensin system activity, damaging the coronary artery walls and predisposing to coronary artery lesions (CAL) [37]. Therefore, there is an inverse correlation between Hb levels and the risk of CAL in KD patients [36]. In this study, Hb levels were significantly lower during the acute phase in patients with CAL. Following IVIG treatment, Hb levels gradually increased, with no significant differences between the two groups during the recovery phase. Hb level can serve as a risk factor for developing CAL during the acute phase of KD but not be a sensitive indicator for evaluating the IVIG treatment efficacy.

CRP and ESR are two classical markers of KD activity and are elevated in patients with KD [12–14]. Interestingly, this study also showed that patients with KD and CAL^+^ had elevated CRP and ESR before and after treatment with IVIG compared with those with CAL^−^. A previous study showed that CRP levels were independently associated with CAL^+^ in children with KD [15]. Still, the previous study showed no differences in ESR between CAL^+^ and CAL^−^ patients, but the previous study also reported no significant differences in white blood cells and platelets between groups, suggesting that the characteristics of the study population were different from the present study. On the other hand, another study reported lower CAL rates in patients with KD and high CRP levels in the acute and convalescence phases [38]. Although ESR and CRP are well-known markers in KD, their relation to CAL^+^ appears variable and would require additional study.

This study also had some limitations. First, this was a retrospective study, and it may have had inaccuracies and inconsistencies in the measurements of blood characteristics due to different people and different machines recording the data. Second, the sample size was small, and the outcome may not have been accurate. This study included confirmed consecutive cases of typical KD and excluded patients with diseases or taking drugs that could affect the results of white blood cells and platelets. Hence, the exclusion criteria were set to exclude the factors that could interfere with the routine blood examination results, thus ensuring the validity and uniformity of the data, but it also reduced the sample size. Finally, the data was retrieved from one hospital, and the extrapolation of the results to others may not have been accurate.

## Conclusions

In conclusion, complete blood count (CBC) is one of the most widely used clinical tests to determine various conditions and diseases due to the reliable results, ease of operation, and affordability. In the context of KD, parameters such as NE, PLR, CRP, ESR, and Hb can serve as indicators for predicting the risk of developing CAL, evaluating treatment efficacy, and assessing prognosis. Therefore, CBC holds significant clinical value in determining KD severity.

## Data Availability

All data generated or analyzed during this study are included in this published article.
